# Ecotoxicological impacts of industrial effluents on irrigation water quality, animal health and the role of calcium alginate in effluents treatment

**DOI:** 10.1007/s10661-022-10216-3

**Published:** 2022-07-15

**Authors:** Hanaa Abdel Atty Zeid, Moustafa Mohsen El-Zayat, Abeer El-Said Abdrabouh

**Affiliations:** 1 Zoology Department, Faculty of Science, Mansoura University, Mansoura, Egypt; 2 Genetic Engineering and Biotechnology Unit, Faculty of Science, Mansoura University, Mansoura, Egypt

**Keywords:** Industrial effluents, Heavy metals, Irrigation indices, Liver, Thyroid gland, Hematology

## Abstract

The effluents discharged from Mansoura Company for Resins and Chemicals Industry were evaluated for drinking and irrigation purposes. Calcium-alginate beads were used for effluents treatment in this study. Young male rats were also allowed to drink effluents at different concentrations (10%, 50%, 100%) and treated 100% effluents with calcium-alginate for 11 weeks. Results indicated high concentrations of some physicochemical parameters and Cd, Co, Fe, Mn, Ni, Pb, and Zn in effluents that exceeded the permissible limits for drinking and irrigation purposes. Treatment by calcium-alginate alleviate heavy metals concentration but did not affect the physicochemical parameters. Depending on effluents concentration, the liver of young male rats showed high accumulation of Fe, Mn, Zn, Pb, Cd, Co, Cu, Cr, and Ni compared to the control group. Serum levels of liver enzymes, total bilirubin significantly increased while total protein, and albumin contents decreased in effluent groups. Liver concentrations of malondialdehyde and protein carbonyl significantly elevated along with significant decrease in superoxide dismutase, catalase, glutathione-S-transferase activities, and glutathione content. Moreover, growth and thyroid hormones were significantly reduced along with significant elevation in thyroid stimulating hormone. This was accompanied by significant decrease in the body weight, especially with 100% effluents concentration compared to control group. Also, histological investigations of both liver and thyroid gland using hematoxylin and eosin showed distortion in the structure of both organs especially with 50% and 100% effluent groups. However, treatment of effluents by calcium-alginate improved these changes. The study revealed that calcium-alginate are effective biosorbents for heavy metals and consequently decrease animal and human health hazards, but further studies are needed to alleviate physicochemical characteristics.

## Introduction

The scarcity of freshwater is a major challenge in Egypt that goes with the increase in population growth (El-Rawy et al., 2020). Water resources used for agricultural activities may be of poor-quality resulting from either natural or anthropogenic contamination or even both (Abugu et al., 2021). It was reported that more than 10% of the global population consume agriculture-based products irrigated by wastewater (Kesari et al., 2021).

The direct disposal of effluents from various industries in a nearby water body without any treatment is a common practice in several countries including Egypt. Most industries do not have a plan for effluent treatment, and even if they have, they are not implemented because of the cost (Barmon et al., 2018; Channa et al., 2020). Unfortunately, the disposed effluents in drains are connected to several canals used for agricultural activities, either for irrigation or watering livestock (Islam et al., 2020). Irrigation with industrial effluents either directly or mixed with canals water exhibited high risk (Ungureanu et al., 2020). The sources of risk are represented in organic, inorganic, and pathogenic contamination (Chaoua et al., 2019). Among various contaminants, heavy metals are known to have slow degradation, and high potential for accumulation and biomagnification causing toxicity through food chains (Tang et al., 2013). Chaoua et al. (2019); Mahfooz et al. (2020) illustrated that because heavy metals are non-biodegradable and have long biological half-life, they can be accumulated at depth of 20 cm of topsoil and absorbed through plant roots. Therefore, human and animal consumption of leafy vegetables expose them to health risk. On the other hand, several studies recommended that application of untreated effluents in irrigation may lead to soil erosion results from increasing soil salinity followed by sodium accumulation. The latter can deteriorate the soil and decrease soil permeability which in turn can reduce the uptake of nutrients by crops from the soil (Kesari et al., 2021; Obasi et al., 2021; Assegide et al., 2022). Measurements of electrical conductivity, pH, total dissolved solids, major anions, and cations in addition to some heavy metals such as Cd, Co, Cr, Cu, Fe, Mn, Ni, Pb, and Zn along with chemical oxygen demand (COD) as an indicator for organic pollution are necessary to evaluate the healthiness of water to be suitable for irrigation and/or drinking (Bauder et al., 2014; Omer, 2019).

In Mansoura city, Mansoura Company for Resins and Chemicals Industry (MRI) is a pioneer in the production of formaldehyde, urea, phenolic resins including molding powder, brake lining, foundry resins, and other chemicals in Egypt, the Middle East, and Africa (El-Agrody et al., 2018). The illegal discharge of large amounts of untreated effluents directly to the nearest water body represents an environmental problem. Several farmers ignore the deleterious effects of these xenobiotics, where water mixed with effluent components may be used for agricultural activities.

The assessment of systemic toxicity of untreated MRI effluents using a mammalian test model will be more relevant to the health of livestock. Biomarkers of systemic toxicity in mammals depend mostly on biochemical, histopathological, and hematological analyses to clarify the possible mechanisms of toxicity and tissue damage after exposure to a mixture of xenobiotics (Adoeoye et al., 2015; Balali-Mood et al., 2021). The liver is the major organ for conjugating and detoxifying any potential toxic substances. It is also essential for intermediary metabolism and the synthesis of several important compounds (Osuala et al., 2014). The concentration of liver enzymes in the blood is an important diagnostic biomarker for assessing liver damage (Talkhan et al., 2016). Several studies have reported that intracellular accumulation of metals stimulates free-radical reactions causing continuous production of reactive oxygen species (ROS) (Elroghy & Yassien, 2021; Balali-Mood et al., 2021; Kim et al., 2021). This results in an imbalance between oxidants and antioxidants leading to lipid peroxidation and protein modification (Espin et al., 2014; Reddy et al., 2015; Balali-Mood et al., 2021).

Moreover, the endocrine system has an important role in the growth and development of the animal body. Previous studies indicated that nickel could affect endocrine organs, causing hormonal disorders (Yang & Ma, 2021). The hypothalamus and pituitary gland are the regulatory centers of the endocrine system. If they are damaged, the regulation of the endocrine axis is disturbed (Aderdara et al., 2019). The anterior pituitary gland secretes growth hormone (GH) and thyroid stimulating hormone (TSH) which are important for growth and development (Yang & Ma, 2021). Very little information is provided about environmental stressors and their effects on the thyroid gland. Thyrotoxicity occurring chemically is accompanied by imbalanced plasma T4 and/or T3 and/or TSH (Buha et al., 2018).

On the other hand, different techniques have been applied for treatment of effluents polluted with heavy metals, including chemical precipitation, coagulation-flocculation, and ion exchange (Renu et al., 2017; Silva et al., 2008; Türkmen et al., 2022). However, usage of metal biosorbents derived from living organisms has been developed for this purpose, especially those derived from seaweeds (macroscopic algae) and alginate derivatives that are widely abundant and less expensive than industrial synthetic adsorbents (Aslam et al., 2021; Hamdy et al., 2018; Qamar et al., 2022). Algins are salts of alginic acid, a natural polymer found in brown algae (*Phaeophyceae)*. This polymer is extracted by treating seaweeds with sodium carbonate solution, where alginic acid is precipitated and sodium salt is formed. In addition, alginate products are known to have high sorption uptake, abundance, high selectivity, and no toxic effects (He & Chen, 2014; Li et al., 2021). Several studies revealed that sodium alginate is an efficient candidate for heavy metals adsorption such as Cu^2+^, Cd^2+^, and Pb^2+^ (Gao et al., 2020; Qamar et al., 2022; Refaay et al., 2022). However, others used alginate in the form of calcium alginate beads because the dry beads have higher sorption capacity per unit mass and are more suitable for practical handling (Aslam et al., 2021; Bilal & Iqbal, 2019; Silva et al., 2008). Therefore, this study aims to evaluate the quality of MRI effluents for irrigation and watering animals through analyzing physicochemical characteristics and heavy metals concentration and comparing them to world health organization (WHO) limits for drinking water and food and agricultural organization (FAO) limits for irrigation water. The study was also extended to use calcium alginate beads in the treatment of effluents. Besides, an in vivo experimental study was designed to mimic the exposure of young cattle in rural areas that receive untreated MRI effluents at different concentrations to examine the effects on the health of young male rats, with reference to the role of alginate in alleviation.

## Materials and methods

### Ecological studies

#### Study area

**Fig. 1:**
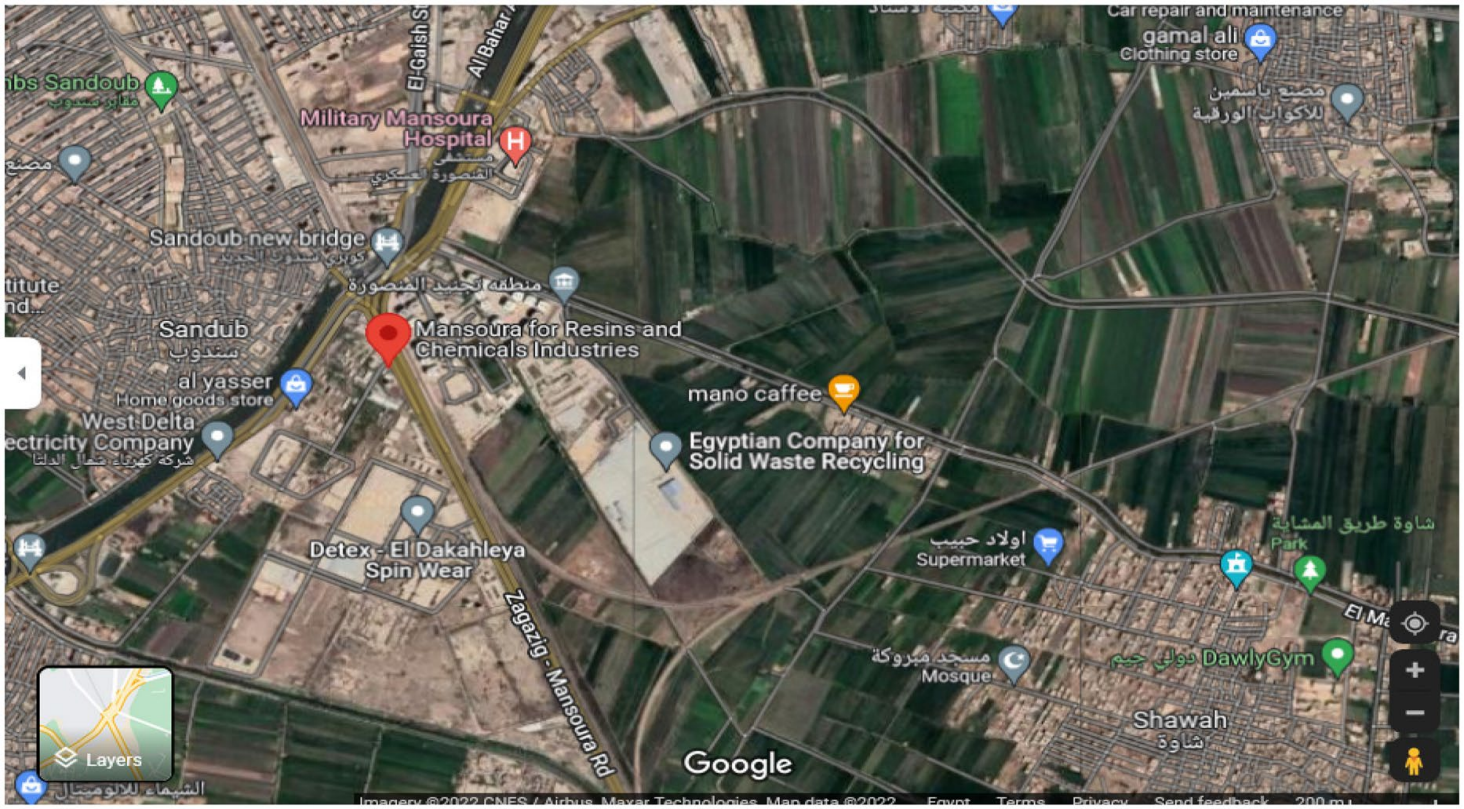
Location of the study area using Google earth

The present study was carried out at the center of Dakahlia Province, precisely near the Mansoura Company for Resins & Chemicals Industry (MRI). This factory is located at Sandoub-Elsinbellaween road in Mansoura city (31° 05′ 00" N, 32° 0′ 48" E), 40 km from Damietta port (Fig. 1). The company pours untreated effluents directly into an El-Mansoura drain that passes through several agricultural villages such as Ezbet Shawwa, Sandoub, Telbana, Miniet Sandoub, Belgai, Ezbet Belgai, and Gemiza Belgai.

#### Effluent sampling

Water samples were collected directly from the discharge point of the effluents of the MRI company, during the period from February to April 2020 (3 samples/month). The nine samples were taken in clean polyethylene bottles of 10 L capacity, where each bottle was rinsed three times before collecting the sample. Approximately 5 ml of concentrated nitric acid was added to the whole sample to prevent heavy metals from precipitating.

#### Field investigations

The hydrogen ion concentration (pH) value of the samples was determined by an electrical-pH meter in the field (Model Corning, NY 14,831 USA). Electrical conductivity (EC) was detected immediately in water samples in the field using a Model C535 multiparameter analyzer.

#### Laboratory investigations

The collected water samples were analyzed at the laboratory of Agricultural Research Center, Mansoura, Egypt. The investigated parameters included total dissolved salts (TDS) which were measured directly using CORNING (Cole –Parmer model Check 90). Bicarbonate (HCO_3_^−^) and sulfates (SO_4_^2−^) were detected according to (Piper, 1947). Chlorides (Cl^−^) were determined using the Meneer method as described in the American Public Health Association (APHA, 1998). Sodium (Na^+^) and potassium (K^+^) were detected by using a flame photometer (Model PH 80B Biologie Spectrophotometer). Calcium (Ca^++^) and magnesium (Mg^++^) were estimated using versenate (EDTA) titration method according to (Jackson, 1973). However, chemical oxygen demand (COD) was determined according to (Rice et al., 2017).

#### Irrigation water quality indices

To evaluate water suitability for irrigation purposes, five parameters were commonly used: the sodium adsorption ratio (SAR) detected from the equation:

SAR =$$\frac{\mathrm{Na}}{\sqrt{\frac{\mathrm{Mg}+\mathrm{Ca}}{2}}}$$ (Richards, 1954).

Kelley’s ratio (KR) which defines the harmful impacts of sodium on irrigation water quality was expressed as:

KR =$$\frac{\mathrm{Na}}{\mathrm{Mg}+\mathrm{Ca}}$$ (Kelley, 1963).

Sodium percentage (Na%) was calculated from:

Na% = $$\frac{\mathrm{Na}+\mathrm{K}}{\mathrm{Ca}+\mathrm{Mg}+\mathrm{Na}+\mathrm{K}}$$ *100 (Wilcox, 1955).

The permeability index (PI) which defines water suitability for the irrigation, index is calculated from:

PI = $$\frac{\mathrm{Na}+\sqrt{\mathrm{HCO}3}}{\mathrm{Ca}+\mathrm{Mg}+\mathrm{Na}}$$ *100 (Doneen, 1964).

The magnesium adsorption ratio (MAR) was calculated as:

MAR = $$\frac{\mathrm{Mg}}{\mathrm{Mg}+\mathrm{Ca}}$$ *100 (Raghunath, 1987).

#### Heavy metals analysis

The MRI effluent samples were analyzed for different heavy metals concentration, including cadmium (Cd), cobalt (Co), chromium (Cr), copper (Cu), iron (Fe), manganese (Mn), nickel (Ni), lead (Pb), and zinc (Zn). Effluent samples were filtered through Whatman No.1 filter paper before analysis. Bi-distilled deionized water was properly used for washing glassware throughout the study. The metals concentration was detected on a Buck Scientific Accusys 211 series “Atomic Absorption Spectrophotometer”, USA, by an air/acetylene flame system according to standard methods provided by APHA (2005) at wavelengths of 228.90 nm, 240.70 nm, 357.90 nm, 324.70 nm, 248.30 nm, 279.50 nm, 341.50 nm, 283.20 nm, and 213.90 nm, respectively. The standard solutions were prepared in range of 0.2–1.6 mg/L for Cd, Mn, and Zn, 0.5–4 mg/L for Cu, Co, Cr, Fe and Ni, and 1–8 mg/L for Pb by diluting stock solutions of standards purchased from Agilent Technologies, Germany. The standards were run after every 15 sample readings to assure more than 95% accuracy.

### Effluents treatment

#### Preparation of Ca-alginate beads

According to EL-Tayieb et al. (2013), sodium alginate powder was mixed with distilled water (3% w/v) with stirring. The alginate gel solution (Cross-Linker solution) was kept in refrigerator for half an hour, then 10 ml was added dropwise into 100 ml of 2% CaCl_2_ solution under gentle stirring at 25 °C. Ca-alginate beads were formed upon contact with the Cross-Linker solution. Beads were left overnight to stabilize, then filtered, washed three times with bi-distilled water, and left to dry to be ready for use in effluents treatment.

#### Application of alginate sorbents on effluent samples

Sorption was performed by adding 100 g dried Ca-alginate beads to a volume of 1000 ml effluents sample in a reactor. Sorption was evolved with the mixture under orbital shaking at 25 °C. After 5 h, volume samples were collected from the reactor and metal concentrations were detected again by atomic absorption spectroscopy, USA by an air/acetylene flame system.

### Animal experimental design

A total of 50 young male Wistar rats (45 ± 5 g) were obtained from the Egyptian Institute for Serological and Vaccine Production, Helwan, Egypt. The rats were allowed to acclimatize for one week to standard conditions of illumination with a 12 h light and dark cycle at 23 ± 3 °C room temperature and 40 ± 5% humidity. Water and food were consumed ad libitum according to the Ethics Committee of Mansoura University, Egypt. The food consisted of corn, soybean pulp, sunflower seed meal, alfalfa pellets, molasses, vitamins, and minerals that are commonly used for poultry farms.

After the acclimation period, rats were randomly divided into five groups (10 rats/each) as follows:

Group 1 was the control (CN) group, which received tap water.

Group 2 received 10% concentration of MRI effluents (10%.EF).

Group 3 received 50% concentration of MRI effluents (50%.EF).

Group 4 received 100% concentration of MRI effluents (100%.EF).

Group 5 received 100% effluents after treatment with Ca-alginate beads (100%.EF + AG) group.

Each group received specific drinking water daily for 11 weeks.

### Animal investigations

#### Body weight changes

The weight of each rat in each group was detected weakly using a digital balance. However, the body weight gain was calculated from the equation:

Body weight gain = Final body weight - initial body weight.

#### Blood sampling

At the end of the experimental period, rats were weighed and sacrificed. From each rat, two blood samples were collected, and the first was received in an EDTA tube to assess hematological indices. However, the second sample was collected in a sterilized tube without anticoagulant and centrifuged at 860 × g for 15 min. Nonhemolyzed sera were separated and kept at − 20 °C until analysis.

#### Hematological indices

Blood samples collected on EDTA were used to determine red blood cells (RBCs) count, hemoglobin (Hb) content, hematocrit (Hct%), mean corpuscular volume (MCV), mean corpuscular hemoglobin (MCH), mean corpuscular hemoglobin concentration (MCHC), white blood cells (WBCs) count, and platelets (PLts) count using a Sysmex Cell Counter (Sysmex, Japan), according to Dacie and Lewis (2001).

#### Serum liver function analysis

Serum alanine aminotransferase (ALT) and serum aspartate aminotransferase (AST) activities were estimated using the enclosed method in Spinreact kits (Ctra Santa Coloma, Spain). However, serum total bilirubin (TB) level and albumin (Alb) content were detected by using the method of Diamond Diagnostics, Co. Kit, Cairo, Egypt. Serum total protein (TP) content was determined using a kit purchased from Bio-Diagnostic Co. Dokki, Giza, Egypt.

#### Serum hormonal analysis

Enzyme-linked immunosorbent assay (ELISA) kits were used to detect serum growth hormone (GH) concentrations in different investigated groups, according to the manufacturer’s protocol of MyBioSource, USA. The levels of serum total triiodothyronine (T3) and thyroxine (T4) were measured according to the method of chemiluminescent enzyme immunoassay enclosed in Siemens Healthcare Diagnostics products LTD- IMMULITE^®^ 2000 systems. An ELISA kit was also used to detect serum thyroid stimulating hormone (TSH) according to the manufacturer’s protocol of LDN, Germany.

#### Tissue sampling

Immediately after collecting blood samples, rats of each group were dissected, the thyroid gland and a part of the right lobe of the liver were separated, for histological studies. However, approximately 0.5 g from the left lobe of liver was taken, homogenized in 5 ml cooled distilled water, and centrifuged at 860 × g for 15 min. The clear supernatant was collected and stored at − 20 °C until analysis of antioxidants and oxidative stress markers. A third sample of the liver tissue was kept at − 20 °C for heavy metal analysis.

#### Liver antioxidants and oxidative stress markers

The liver superoxide dismutase (SOD) and catalase (CAT) activities, in addition to reduced glutathione (GSH) content and malondialdehyde (MDA) concentration, were estimated using the specific enclosed methods of Biodiagnostic Co. Dokki, Giza, Egypt. However, an ELISA kit was used to determine glutathione S-transferase (GST) concentration in the liver according to the manufacturer’s protocol of Cusabio, USA. Moreover, the protein carbonyl (PC) level in the liver was assessed according to the manufacturer’s protocol enclosed in the Cayman Chemical Kit (Michigan, USA).

#### Heavy metal concentrations in liver

Nitric-sulfuric-perchloric acid digestion is an approach that is partly modified from that of Tinggi et al. (1997). One gram of each sample was weighed into a conical flask, and 10 ml of concentrated HNO_3_ was added. The conical flask was allowed to heat on a hot plate at 95 °C until white fumes evolved and then left to cool at room temperature. After cooling, 3 ml of concentrated H_2_SO_4_ was added and then heated until the release of orange fumes. The flask was allowed to cool again at room temperature, and then 2 ml of HClO_4_ was added and heated until the solution was reduced to approximately 5 ml. Each digested sample was filtered through 0.45-µ Millipore membrane filter paper diluted to 50 ml with deionized water in a volumetric flask and analyzed using a Buck Scientific atomic absorption spectrometer (model accusys 211, USA) equipped with an air/acetylene flame and hollow cathode lamps for the analyzed elements.

#### Histological studies

Evaluation of the structural changes in the liver and thyroid gland via light microscopy was achieved by hematoxylin and eosin (H&E) staining. According to the method of Kiernan et al. (2008), the tissues were immediately fixed in 10% formol saline after dissection, embedded in paraffin, sectioned at 5-μm thickness, and stained.

## Statistical analysis

The data were analyzed using the GraphPad Prism software program (v 5.04 GraphPad Software Inc., La Jolla, CA) using one-way ANOVA followed by Tukey test. All the results were recorded as the mean ± SD. The Pearson correlation coefficient was used to estimate the correlations between physicochemical characteristics and/or the selected heavy metals in untreated and treated effluent samples. Statistically significant data were considered at *p* < 0.05.

## Results

### Ecological studies

#### Physicochemical characteristics of effluent samples

The appropriateness of effluents for drinking and irrigation purposes was evaluated through physicochemical characteristics. A statistical summary of the physicochemical parameters of the effluent samples is listed in Table 1 as follows:

##### Electrical conductivity

The maximum and minimum electrical conductivity (EC) values were 3.56 and 2.06 dS/m for untreated MRI effluents (EF) and 3.87 and 3.17 dS/m for effluents treated with Ca-alginate (EF + AG), respectively. Moreover, by comparing the mean EC values of different effluent samples to WHO limits for drinking water and FAO limits for irrigation water, the mean EC values (2.802 ± 0.59 and 3.600 ± 0.30) dS/m of both untreated (EF) and treated effluents (EF + AG), respectively exceeded the WHO limits. However, EF samples were within FAO limits for irrigation water, but EF + AG samples were greater than the FAO limits.

##### pH value

The pH values of untreated MRI effluent samples, as well as treated effluents, exhibited mostly acidic features. The maximum and minimum recorded pH values were (8.33, 4.43) and (8.11, 4.71), respectively. The mean pH value for the untreated samples (5.71 ± 1.77) was found to be below the permissible limits recommended by the WHO for drinking water and FAO for irrigating water. However, that of treated effluent samples (6.46 ± 1.59) was within these limits.

##### Total dissolved solids

Total dissolved solids (TDS) values showed maximum and minimum values (2918.42 and 1318.46 mg/L for MRI effluents and 2368.0 and 1248.0 mg/L for effluents treated with Ca-alginate, respectively). The average of both samples (1921 ± 618.3 and 1992 ± 518.1) mg/L exceeded the permissible drinking water limit recommended by the WHO but was within the limits of the FAO for irrigation water.

##### Anionic composition

The maximum and minimum HCO_3_^−^ contents of the effluent samples were 162.5 and 94.79 mg/L and 189.59 and 67.1 mg/L for EF and EF + AG, respectively. However, the maximum and minimum values of the Cl^−^ content of the samples were 817.6 and 483.9 mg/L and 984.4 and 631.0 mg/L for EF and EF + AG, respectively. The SO_4_^−−^ content of the investigated water samples varied between 621.6 and 472.1 mg/L for EF and 519.7 and 326.1 mg/L for EF + AG. The mean values of HCO_3_^−^ in EF and EF + AG (125.3 ± 30.03, 105.0 ± 57.85) were within the permissible limits of WHO for drinking water and FAO for irrigation water. However, the mean values of both Cl^−^ and SO_4_^−−^ in EF and EF + AG (634.0 ± 124.3, 838.0 ± 148.5 and 515.3 ± 71.11, 431.2 ± 69.92, respectively) exceeded WHO drinking water limits in addition to FAO limits for SO_4_^−−^ values.

##### Cationic composition

The maximum and minimum Na^+^ concentrations were 657.2 and 434.65 mg/L for EF samples and 830.49 and 418.77 mg/L for EF + AG, respectively. However, K^+^ concentrations showed maximum and minimum values (9.0, 5.0) and (8.0, 5.50) mg/L for EF and EF + AG, respectively. Moreover, the maximum and minimum Ca^++^ concentrations of the effluent samples were (26.40, 17.60) and (30.80, 17.45) mg/L for EF and EF + AG, respectively. The Mg^++^ concentrations of the effluent samples ranged between (16.75, 10.18) and (16.65, 10.08) mg/L for EF and EF + AG, respectively. Furthermore, for both untreated and treated effluents, the mean Na^+^ concentrations (551.0 ± 104.8 and 681.1 ± 185.2) respectively increased above the WHO drinking water limits but were within the FAO irrigation limits. The mean concentrations of K^+^ ions in both samples (6.63 ± 1.7 and 6.90 ± 0.96) were within WHO and exceeded FAO limits. However, the mean values of Ca^++^ (20.24 ± 3.94, 22.89 ± 5.72) and Mg^++^ (13.19 ± 3.103, 13.06 ± 2.71) in EF and EF + AG samples, respectively, were within both WHO drinking water and FAO irrigation water limits.

##### Chemical oxygen demand

The chemical oxygen demand (COD) values of the collected effluent samples showed maximum and minimum values between (944.0, 859.0) for EF and (912.0, 818.0) mg/L for EF + AG. Moreover, the COD mean values (889.5 ± 37.37 and 878.5 ± 41.62) for EF and EF + AG, respectively, exceeded the permissible drinking water levels of the WHO. However, COD limits for irrigation water were not included in FAO standards.

It should also be mentioned that the treatment of effluents with Ca-alginate did not statistically show any significant differences in the estimated physicochemical parameters compared to untreated effluent samples.

##### Irrigation water indices

Table 2 shows the maximum and minimum values of irrigation indices, where SAR was 154.0 and 116.6 for EF and 183.9 and 126.8 for EF + AG samples. The KR values were within (19.14, 12.71) and (24.68, 18.22) for EF and EF + AG, respectively. However, Na% in MRI effluent samples varied between (95.12, 92.83)% and (95.29, 91.66) % for EF + AG samples. The PI values showed maximum and minimum values between 96.63 and 94.66 for the EF and 96.24 and 93.33 for the EF + AG samples. Finally, MAR % lies between 45.65 and 33.41 for EF and 37.07 and 28.93 for EF + AG. According to water quality criteria, the mean values of all the investigated irrigation indices referred to the unsuitability of discharged effluents for irrigation purposes, except MAR%.

**Table 1:** Physicochemical characteristics of untreated and treated MRI effluent samples

**Parameter**	**EF**			**EF + AG**			**WHO** (2008)	**FAO** Ayers and Westcot (1994)
	Max	Min	Mean ± SD	Max	Min	Mean ± SD		
**EC (dS/m)**	3.56	2.06	2.802 ± 0.59	3.87	3.17	3.600 ± 0.30	0.4	0.7–3 Ayers and Westcot (1985)
**pH-value**	8.33	4.43	5.71 ± 1.77	8.11	4.71	6.46 ± 1.59	6.5 –8.5	6.5 – 8.4
**TDS (mg/L)**	2918.42	1318.46	1921 ± 618.3	2368.0	1248.0	1992 ± 518.1	500	2000
**HCO**_**3**_^**−**^** (mg/L)**	162.5	94.79	125.3 ± 30.03	189.59	67.1	105.0 ± 57.85	150	600
**Cl**^**−**^** (mg/L)**	817.6	483.9	634.0 ± 124.3	984.4	631.0	838.0 ± 148.5	250	1000
**SO**_**4**_^**−−**^** (mg/L)**	621.6	472.1	515.3 ± 71.11	519.7	326.1	431.2 ± 69.92	250	400
**Na**^**+**^** (mg/L)**	657.2	434.65	551.0 ± 104.8	830.49	418.77	681.1 ± 185.2	200	920
**K**^**+**^** (mg/L)**	9.0	5.0	6.63 ± 1.7	8.0	5.50	6.90 ± 0.96	12	2
**Ca**^**++**^** (mg/L)**	26.40	17.60	20.24 ± 3.94	30.80	17.45	22.89 ± 5.72	100	60
**Mg**^**++**^** (mg/L)**	16.75	10.18	13.19 ± 3.103	16.65	10.08	13.06 ± 2.71	120	1063
**COD (mg/L)**	944.0	859.0	889.5 ± 37.37	912.0	818.0	878.5 ± 41.62	250	-

Data are presented as maximum, minimum, and mean ± SD of different effluent samples; *EF*, untreated MRI effluents; *EF* + *AG*, treated MRI effluents with Ca-alginate

a: Significant change in comparing the EF group with the EF + AG group (*p* < 0.05), where no significant changes were observed

WHO represents World Health Organization limits for drinking water. FAO represents the Food and Agriculture Organization of the United Nations limits for irrigation water

**Table 2:** Irrigation water indices in untreated and treated MRI effluent samples

Parameters	EF			EF + AG			Water quality criteria
	Max	Min	Mean ± SD	Max	Min	Mean ± SD	
**SAR**	154.0	116.6	135.6 ± 18.84	183.9	126.8	166.9 ± 26.88	< 10, excellent, 10–18 good, 18–26 doubtful, > 26 unsuitable Richards (1954)
**KR (meq/l)**	19.14	12.71	16.26 ± 2.77	24.68	18.22	20.56 ± 2.85	< 1 good, > 1 bad Kelley (1963)
**Na (%)**	95.12	92.83	94.17 ± 0.99	95.29	91.66	93.96 ± 1.68	< 20 excellent, 20–40 good, 40–60 permissible, 60–80 doubtful, > 80 unsuitable Wilcox (1955)
**PI (meq/l)**	96.63	94.66	95.62 ± 0.90	96.24	93.33	95.39 ± 1.39	< 20 excellent, 20–40 good, 40–80 injurious, > 80 unsatisfactory Doneen (1964)
**MAR (%)**	45.65	33.41	40.40 ± 5.34	37.07	28.93	33.89 ± 3.79	< 50 suitable, > 50 unsuitable Raghunath (1987)

Data are presented as maximum, minimum, and mean ± SD of different water samples; *EF*, untreated MRI effluents; *EF* + *AG*, treated effluents with Ca-alginate

##### Heavy metals in effluent samples

Table 3 illustrates the maximum and minimum concentrations of heavy metals (Cd, Co, Cr, Cu, Fe, Mn, Ni, Pb, and Zn) in MRI effluents (EF) and effluents treated with alginate (EF + AG). The mean concentrations of heavy metals in EF samples were arranged descendingly as Mn, Fe, Zn, Cd, Co, Ni, Pb, Cu, and Cr. Most of the detected heavy metals in MRI effluents exceeded the WHO limits for drinking water, where Co (0.139 ± 0.015) mg/L, Fe (1.235 ± 0.520) mg/L, Mn (1.681 ± 0.496) mg/L, Cd (0.247 ± 0.071) mg/L, Pb (0.120 ± 0.008) mg/L, Ni (0.122 ± 0.034) mg/L, and Zn (0.945 ± 0.113) mg/L compared to (0.04), (0.3), (0.4), (0.003), (0.01), (0.07), and (0.2) mg/L WHO limits, respectively. However, according to FAO irrigation water limits, MRI effluents showed unsuitable concentrations of Co, Mn, and Cd compared to the (0.05), (0.2), and (0.01)mg/L FAO limits. On the other hand, treatment of MRI effluents with alginate showed a significant decrease in all the detected heavy metals compared to untreated effluents. Although this decrease was not below standard limits, it was greatly near their values in most of the investigated metals, where descending arrangement of metals was Mn, Fe, Zn, Cd, Pb, Ni, Co, Cu, and Cr.

**Table 3:** Concentration of heavy metals in untreated and treated MRI effluent samples

**Heavy meta**l**s** **(ppm)**	**EF**			**EF + AG**			**WHO** **(ppm)** (2011, 2017)	**FAO** **(ppm)** Ayers and Westcot (1985, 1994)
	Max	Min	Mean ± SD	Max	Min	Mean ± SD		
**Cd**	0.364	0.190	0.247 ± 0.071	0.190	0.131	0.167 ^**a**^ ± 0.024	0.003	0.01
**Co**	0.165	0.129	0.139 ± 0.015	0.086	0.051	0.067 ^**a**^ ± 0.015	0.04	0.05
**Cr**	0.053	0.028	0.036 ± 0.012	0.023	0.019	0.021 ^**a**^ ± 0.002	0.05	0.1
**Cu**	0.079	0.055	0.068 ± 0.010	0.053	0.033	0.039 ^**a**^ ± 0.008	2	0.2
**Fe**	2.053	0.773	1.235 ± 0.520	0.736	0.348	0.564 ^**a**^ ± 0.156	0.3	5
**Mn**	2.515	1.318	1.681 ± 0.496	1.302	0.923	1.091 ^**a**^ ± 0.135	0.4	0.2
**Ni**	0.176	0.093	0.122 ± 0.034	0.092	0.054	0.071 ^**a**^ ± 0.018	0.07	0.2
**Pb**	0.133	0.110	0.120 ± 0.008	0.094	0.064	0.077 ^**a**^ ± 0.014	0.01	5
**Zn**	1.074	0.817	0.945 ± 0.113	0.612	0.467	0.539 ^**a**^ ± 0.055	0.2	5

Data are presented as maximum, minimum, and mean ± SD of different effluent samples; *EF*, untreated MRI effluents; *EF* + *AG*, treated MRI effluents with Ca-alginate

a: Significant change in comparing the EF group with the EF + AG group (*p* < 0.05)

WHO represents World Health Organization limits for drinking water. FAO represents the Food and Agriculture Organization of the United Nations limits for irrigation water

##### Correlation analysis

The Pearson correlation coefficient matrix (r) was used to determine the interaction between physicochemical characteristics and heavy metal concentrations, as well as each other in untreated and treated MRI effluent samples (Table 4). The physicochemical characteristics of effluent samples showed significant positive correlations between Cl^−^/EC, Ca^++^/EC, Ca^++^/TDS, and Ca^++^/Cl^−^. However, the estimated heavy metals in effluent samples exhibited positive and negative but nonsignificant correlations with the detected physicochemical parameters. This could be seen for example, in pairs of Cr/pH (r = 0.229), Fe/Cl^−^ (r = 0.030), Mn/ SO_4_^−−^ (r = 0.186), Co/Cl^−^(r =  − 0.364), Pb/Na^+^(r =  − 0.149), Cd/Mg^++^ (r =  − 0.082), and Ni/COD (r =  − 0.203). On the other hand, the elemental pairs showed significant positive correlation between all investigated metals.

**Table 4:** Pearson correlation coefficient matrix (r) showing the interaction between heavy metal concentrations and physicochemical characteristics in untreated and treated MRI effluent samples

	**pH**	**EC**	**TDS**	**HCO**_**3**_^**−**^	**Cl**^**−**^	**SO**_**4**_^**—**^	**Na**^**+**^	**K**^**+**^	**Mg**^**++**^	**Ca**^**++**^	**COD**	**Cu**	**Co**	**Cr**	**Fe**	**Mn**	**Cd**	**Pb**	**Ni**	**Zn**
pH																				
EC	− 0.164																			
TDS	− 0.052	0.587																		
HCO_3_^−^	− 0.021	0.069	0.554																	
Cl^−^	− 0.070	** 0.825	0.410	0.040																
SO_4_^—^	− 0.334	− 0.036	0.167	0.288	− 0.143															
Na^+^	0.600	− 0.160	− 0.240	− 0.500	0.255	− 0.376														
K^+^	− 0.257	− 0.043	0.114	− 0.156	− 0.065	− 0.529	− 0.006													
Mg^++^	− 0.588	− 0.040	0.156	0.477	0.076	0.211	− 0.359	0.452												
Ca^++^	− 0.208	** 0.814	* 0.668	0.137	*** 0.900	− 0.176	0.126	0.178	0.140											
COD	0.041	− 0.409	− 0.625	− 0.588	− 0.180	0.399	0.470	− 0.400	− 0.254	− 0.417										
Cu	− 0.149	− 0.290	0.292	0.252	− 0.223	0.301	− 0.081	0.129	0.157	0.148	0.138									
Co	0.019	− 0.421	0.213	0.237	− 0.364	0.117	− 0.024	0.130	− 0.034	− 0.096	0.056	*** 0.954								
Cr	0.229	0.060	0.579	0.420	0.003	0.207	− 0.080	− 0.092	− 0.147	0.204	− 0.164	** 0.811	** 0.806							
Fe	0.152	− 0.001	0.514	0.424	0.030	0.250	− 0.031	− 0.045	0.006	0.298	− 0.063	*** 0.897	** 0.835	*** 0.976						
Mn	0.243	0.023	0.570	0.365	0.033	0.186	0.048	− 0.015	− 0.088	0.315	− 0.100	** 0.863	** 0.821	*** 0.985	*** 0.987					
Cd	0.210	0.112	0.591	0.399	0.085	0.137	− 0.039	0.003	− 0.082	0.370	− 0.193	** 0.843	* 0.788	*** 0.990	*** 0.984	*** 0.988				
Pb	− 0.128	− 0.371	0.287	0.366	− 0.316	0.165	− 0.149	0.139	0.095	0.129	− 0.041	*** 0.958	*** 0.980	* 0.770	** 0.834	** 0.808	** 0.797			
Ni	0.040	− 0.005	0.571	0.488	0.031	0.167	− 0.084	0.083	0.088	0.366	− 0.203	*** 0.921	** 0.878	*** 0.952	*** 0.978	*** 0.967	*** 0.968	*** 0.897		
Zn	− 0.032	− 0.410	0.203	0.279	− 0.372	0.261	− 0.115	0.114	0.113	0.002	0.114	*** 0.981	*** 0.970	** 0.815	*** 0.890	** 0.855	** 0.837	*** 0.953	*** 0.900	

### Animal studies

#### Heavy metal concentrations in the liver of different investigated groups

The obtained data in Table 5 show a significant increase in the accumulation of heavy metals (Cd, Co, Cr, Cu, Fe, Mn, Ni, Pb, and Zn) in the liver of MRI effluent groups dependent on effluent concentration compared to the control (CN) group. However, Co and Cr in 50%.EF group in addition to Mn in 10%.EF group were nonsignificant compared to CN group. The metals concentration was significantly decreased with 100%EF treatment by Ca-alginate. This reduction was significant in all detected metals compared to 100%EF group, and mostly with 10% and 50% EF groups. The accumulation of heavy metals in the liver tissues of the investigated groups was descendingly arranged in the following order: (Mn, Co, Cu, Cd, Fe, Pb, Zn, Cr, Ni) for the CN group, (Fe, Mn, Zn, Pb, Cd, Cu, Co, Ni, Cr) for 10%EF group, (Fe, Mn, Zn, Pb, Cd, Co, Cu, Ni, Cr) for 50%EF group, (Fe, Mn, Zn, Pb, Cd, Co, Cu, Cr, Ni) for 100%EF group, and (Mn, Zn, Cd, Fe, Co, Pb, Cu, Ni, Cr) for 100%.EF + AG group. Moreover, by comparing 100%.EF + AG group to CN one, heavy metals concentration showed nonsignificant difference except (Cd) showed significant increase compared to CN.

**Table 5:** Heavy metal concentrations in the liver of the control and different investigated rat groups

**Group**	**CN**	**10% EF**	**50% EF**	**100%.EF**	**100% EF + AG**
**Metals (ppm)**					
**Cd**	0.0016 ± 0.0003	0.0131 ^a^ ± 0.0026	0.018 ^ab^ ± 0.002	0.037 ^ab^ ± 0.004	0.0096 ^a^ ± 0.0006
**Co**	0.0066 ± 0.0007	0.0103 ± 0.003	0.0123 ± 0.003	0.0353 ^ab^ ± 0.004	0.0047 ± 0.004
**Cr**	0.00031 ± 0.0001	0.0022 ± 0.0002	0.0028 ± 0.0009	0.0086 ^ab^ ± 0.0043	0.00033 ± 0.0001
**Cu**	0.005 ± 0.001	0.0111 ^ab^ ± 0.003	0.0115 ^ab^ ± 0.0019	0.0261 ^ab^ ± 0.003	0.003 ± 0.005
**Fe**	0.0013 ± 0.0003	0.0823 ^ab^ ± 0.011	0.125 ^ab^ ± 0.041	0.143 ^ab^ ± 0.021	0.0077 ± 0.003
**Mn**	0.0148 ± 0.0007	0.0512 ± 0.007	0.084 ^ab^ ± 0.28	0.090 ^ab^ ± 0.015	0.044 ± 0.005
**Ni**	0.0003 ± 0.0001	0.0034^ab^ ± 0.0008	0.0037^ab^ ± 0.0009	0.0076 ^ab^ ± 0.002	0.0005 ± 0.0001
**Pb**	0.0012 ± 0.0001	0.016 ^ab^ ± 0.005	0.0196 ^ab^ ± 0.003	0.042 ^ab^ ± 0.007	0.003 ± 0.0002
**Zn**	0.0011 ± 0.0006	0.0281 ^a^ ± 0.0028	0.0378 ^ab^ ± 0.013	0.050 ^ab^ ± 0.006	0.0125 ± 0.0009

Data are presented as the mean ± SD. *CN*, control tap water; 10%, 50%, and 100%.EF, concentrations of MRI effluents; 100%.EF + AG, 100% effluents treated with Ca-alginate

a: Significant change in comparing different groups with the CN group (*p* < 0.05)

b: Significant change in comparing EF groups with 100%.EF + AG group (*p* < 0.05)

#### Liver function

**Fig. 2:**
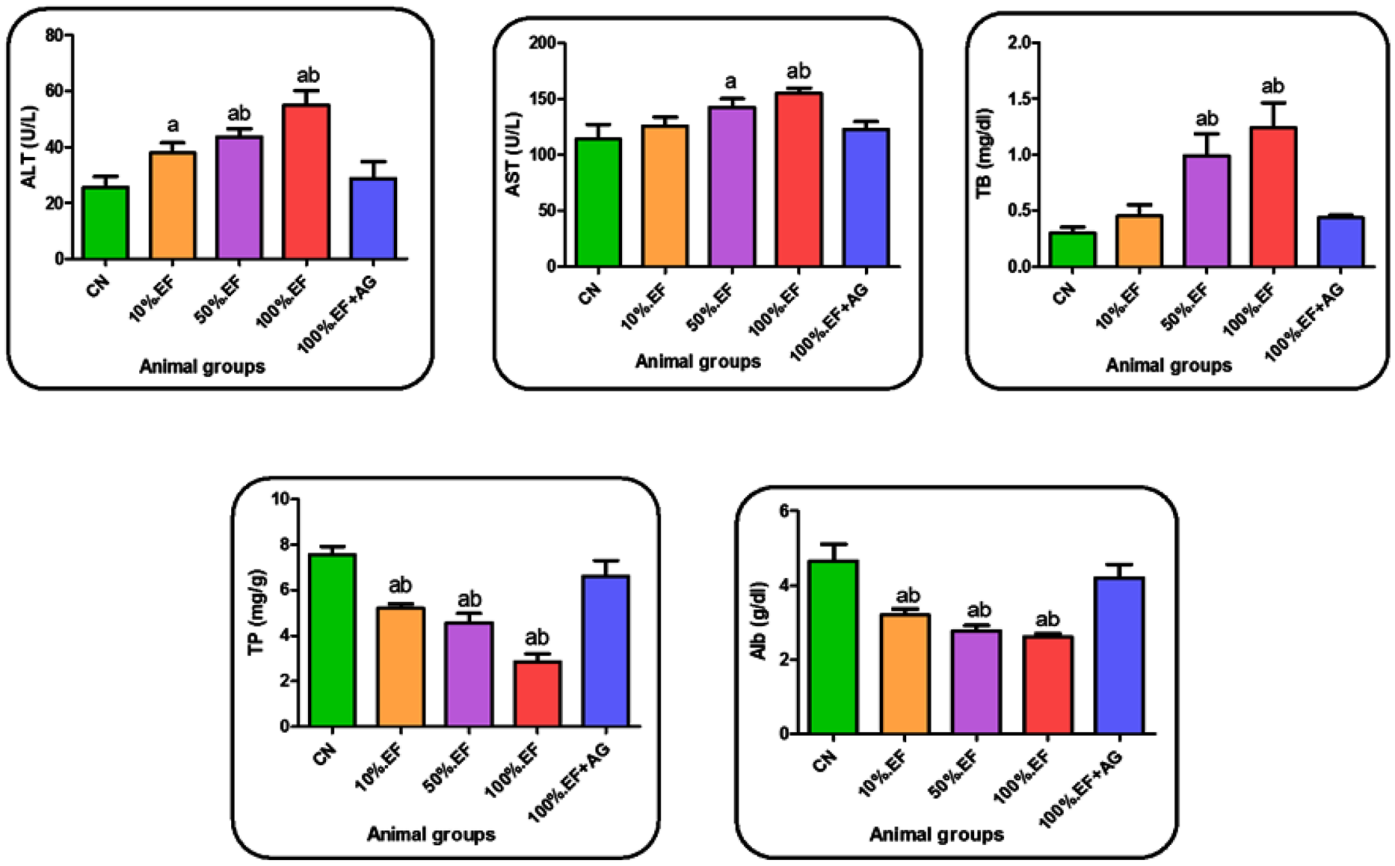
Liver function parameters in the serum of the control and different investigated rat groups. Data are presented as the mean ± SD. CN, control tap water; 10%, 50%, an 100%.EF, concentrations of MRI effluents; 100%.EF + AG, 100% effluents treated with Ca-alginate. **a**: significant change in comparing different groups with the CN group (*p* < 0.05). **b**: significant change in comparing EF groups with 100%.EF + AG group (*p* < 0.05)

The present study showed significant changes in the investigated parameters along all MRI effluent concentrations. ALT, AST, and TB increased significantly especially at 50%EF and 100%EF groups compared to the control group. However, TP and Alb decreased significantly with all the studied concentrations of MRI effluents. Treatment of 100%EF samples with alginate (100%EF + AG) showed significant improvement in liver function parameters represented in significant decrease in ALT, AST, and TB along with an obvious increase in TP and Alb contents compared to all effluent concentration groups as shown in (Fig. 2).

#### Liver antioxidants and oxidative stress markers

**Fig. 3:**
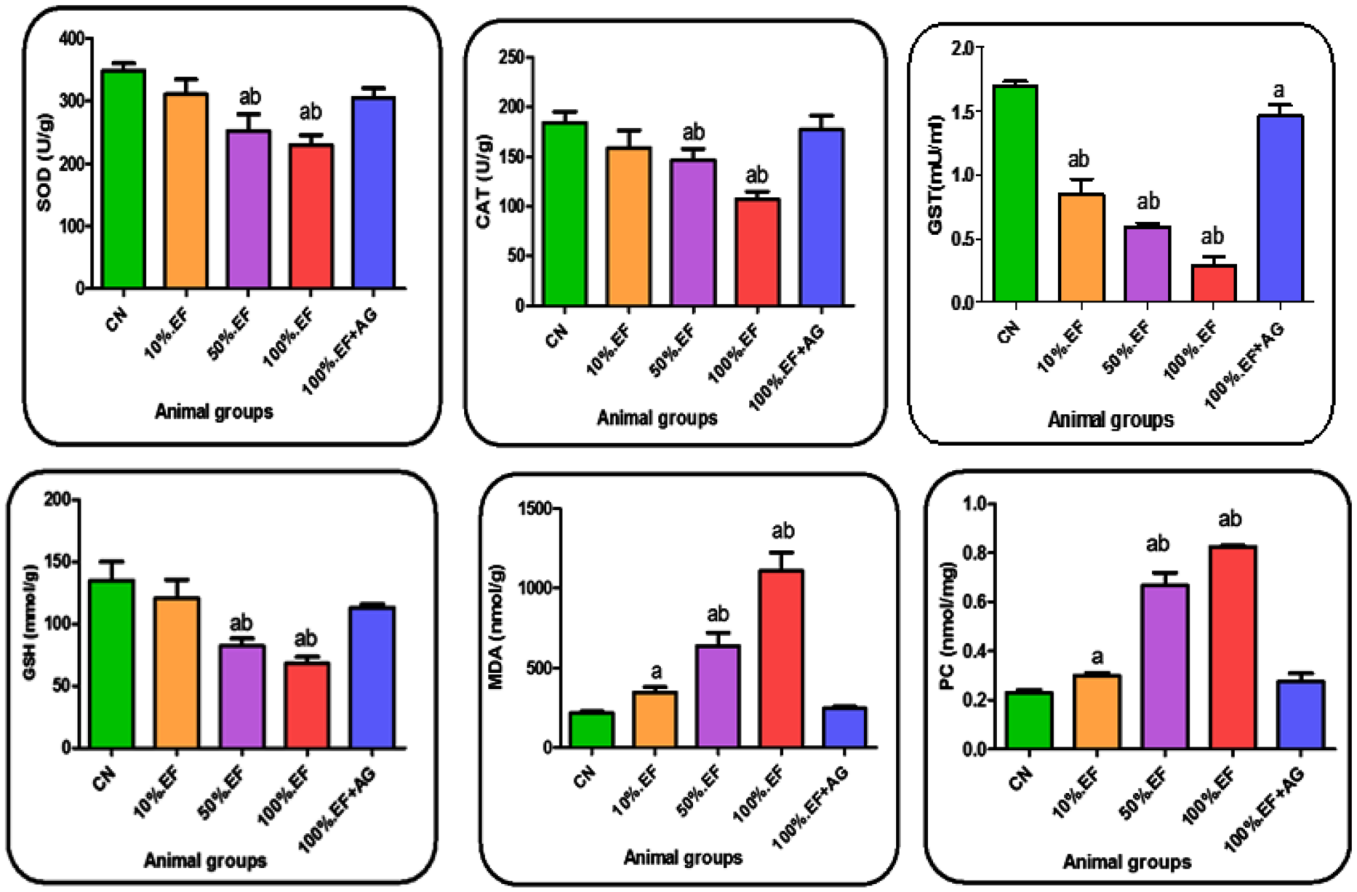
Liver oxidative stress markers and antioxidants in the control and different investigated rat groups. Data are presented as the mean ± SD. CN, control tap water; 10%, 50%, and 100%.EF, concentrations of MRI effluents; 100%.EF + AG, 100% effluents treated with Ca-alginate. **a**: significant change in comparing different groups with the CN group (*p* < 0.05). **b**: significant change in comparing EF groups with 100%.EF + AG group (*p* < 0.05)

The obtained data showed obviously decreased liver antioxidant activities of CAT, SOD, GST, and GSH content with increasing concentrations of MRI effluents compared to the control group. This decrease was mostly significant with 50%.EF and 100%.EF groups. On the other hand, oxidative stress markers (MDA and PC) showed significant increase with drinking different concentrations of MRI effluent groups compared to control. Treatment with Ca-alginate exhibited a significant increase in the studied antioxidants in the liver of 100%.EF + AG rat group which also exhibited a significant decrease in MDA and PC compared to the 50%EF and 100%.EF groups (Fig. 3).

#### Growth and thyroid hormones

To follow the rate of growth experimentally, Table 6 shows the effect of consumption of MRI effluents at different concentrations (10%, 50%, and 100%) on the levels of growth and thyroid hormones. The results exhibited a significant decrease in GH, T3, and T4 hormones, as well as significantly elevated TSH levels with increasing effluent concentrations compared to the control group. Moreover, the rat group that received 100% EF + AG showed a significant increase in GH, T3, and T4 levels along with a decrease in TSH levels compared to all effluent groups.

**Table 6:** Growth and thyroid hormones in the control and different investigated rat groups

**Groups**	**CN**	**10%.EF**	**50%.EF**	**100%.EF**	**100%.EF + AG**
**Parameters**					
**GH (pg/ml)**	2.62 ± 0.29	1.76^**a**^ ± 0.15	1.41^**ab**^ ± 0.19	0.86^**ab**^ ± 0.13	1.97^**a**^ ± 0.22
**T3 (ng/dL)**	142.2 ± 13.71	110.8^**ab**^ ± 17.79	84.17^**ab**^ ± 8.750	46.73^**ab**^ ± 3.001	132.5 ± 10.76
**T4 (µg/dL) **	8.360 ± 0.861	6.667^**ab**^ ± 0.502	5.600^**ab**^ ± 0.529	4.038^**ab**^ ± 0.308	7.942 ± 0.255
**TSH (µIU/mL)**	3.200 ± 0.141	6.245^**ab**^ ± 0.297	8.267^**ab**^ ± 0.708	12.53^**ab**^ ± 1.105	4.233 ± 0.701

Data are presented as the mean ± SD. *CN.0*, control tap water; 10%, 50% and 100%.EF, concentrations of MRI effluents; 100%.EF + AG, 100% effluents treated with Ca-alginate

a: Significant change in comparing different groups with the CN group (*p* < 0.05)

b: Significant change in comparing EF groups with 100%.EF + AG group (*p* < 0.05)

#### The body weight changes

**Fig. 4:**
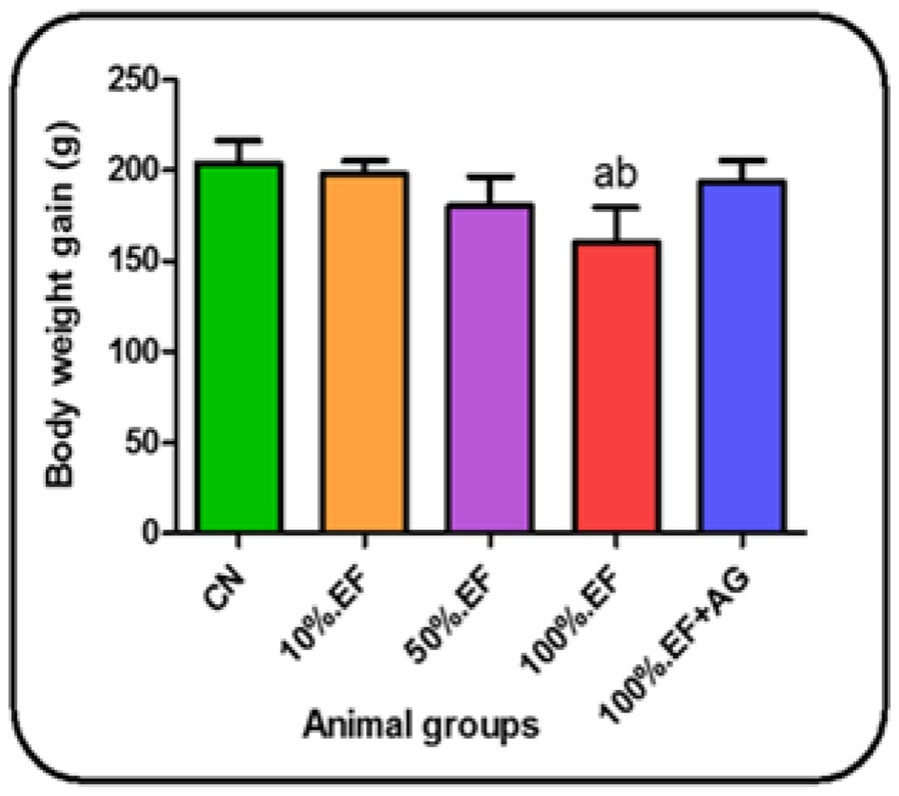
Body weight gain (g) in the control and different investigated rat groups. Data are presented as the mean ± SD. CN, control tap water; 10%, 50%, and 100%.EF, concentrations of MRI effluents; 100%.EF + AG, 100% effluents treated with Ca-alginate. **a**: significant change in comparing different groups with the CN group (*p* < 0.05). **b**: significant change in comparing EF groups with 100%.EF + AG group (*p* < 0.05)

The present results illustrated in Table 7 recorded a decrease in the body weight of rat groups that received different effluent concentrations. This decrease appeared nonsignificant until the sixth week of effluent consumption compared to the control group. However, from the seventh week until the end of the experiment (the eleventh week), the body weight was significantly decreased, especially at 100%.EF group compared to the control group. Moreover, treatment of effluent by Ca-alginate (100%.EF + AG) group significantly increased the body weight compared to untreated 100%.EF group. Accordingly, the body weight gain in all EF groups decreased depending on EF concentration compared to the control group. This decrease was significantly observed, especially at 100%EF group, while treated group (100%.EF + AG) showed a significant increase in body weight gain compared to untreated 100%EF group (Fig. 4).

**Table 7:** Body weight changes in the control and different investigated rat groups

**Weeks**	**CN**	**10%.EF**	**50%.EF**	**100%.EF**	**100%.EF + AG**
**Zero week**	49.13 ± 6.03	49.0 ± 9.0	49.57 ± 4.79	49.0 ± 7.5	49.89 ± 5.21
**1st week**	51 ± 10.2	51.22 ± 8.614	50.9 ± 12.19	50.5 ± 4.96	51.78 ± 8.3
**2nd week**	53.86 ± 7.63	50.0 ± 7.83	51.88 ± 7.59	47.89 ± 5.93	52.43 ± 9.2
**3rd week**	54.5 ± 9.86	51.44 ± 8.69	51.50 ± 7.87	48.89 ± 5.06	54.44 ± 6.6
**4th week**	56.57 ± 9.85	54.0 ± 4.32	53.0 ± 5.48	50.5 ± 4.84	57.38 ± 8.07
**5th week**	58.57 ± 6.53	57.86 ± 8.47	56.29 ± 3.73	52.38 ± 6.28	59.43 ± 9.07
**6th week**	106.4 ± 12.43	103.7 ± 14.86	101.0 ± 13.18	93.7 ± 11.9	108.0 ± 6.07
**7th week**	146.1 ± 15.12	141.7 ± 9.77	138.9 ± 13.53	119.5 ^**ab**^ ± 13.66	143.7 ± 16.53
**8th week**	188 ± 10.33	178.5 ± 7.53	170.1 ± 15.68	151.3 ^**ab**^ ± 14.5	190.0 ± 17.25
**9th week**	203.7 16.78 ±	199.3 9.12 ±	192.0 15.41 ±	173.5 ^**ab**^ 14.38 ±	200.5 ± 14.98
**10th week**	225.7 ± 21.75	210.7 ± 12.15	207.2 ± 12.15	185.7 ^**ab**^ ± 10.17	212.2 ± 12.14
**11th week**	247.2 ± 18.71	240.0 ± 9.83	224.8 ± 15.8	206.8 ^**ab**^ ± 10.83	230.5 ± 10.83

Data are presented as the mean ± SD. *CN*, control tap water; 10%, 50%, and 100%.EF, concentrations of MRI effluents; 100%.EF + AG, 100% effluents treated with Ca-alginate

a: Significant change in comparing different groups with the CN group (*p* < 0.05)

b: Significant change in comparing EF groups with 100%.EF + AG group (*p* < 0.05)

#### Hematological indices

The investigated hematological parameters (RBCs count, Hb content, Hct%, MCV, MCH, MCHC, and WBCs count) decreased significantly with increasing concentrations of MRI effluents in drinking water, especially at 50% and 100% concentrations. However, the pLts count increased significantly at the same concentrations. On the other hand, 100%EF + AG group showed significant improvement in hematological indices compared to the untreated effluent group, especially in RBCs, Hb, Hct, MCV, and MCHC (Table 8).

**Table 8:** Hematological indices in the control and different investigated rat groups

**Groups**	**CN**	**10%.EF**	**50%.EF**	**100%.EF**	**100%.EF + AG**
**Parameters**					
**RBCs (10**^**6**^**/µL)**	7.51	7.34^**b**^	6.70^**ab**^	6.54^**ab**^	8.13
	± 0.39	± 0.62	± 0.24	± 0.41	± 0.31
**Hb (g/dl)**	14.43	13.8	12.97^**ab**^	11.72^**ab**^	14.87
	± 0.11	± 1.05	± 0.66	± 0.85	± 0.36
**Hct (%)**	44.97	42.7	38.73^**ab**^	37.73^**ab**^	47.67
	± 3.21	± 0.80	± 2.30	± 1.37	± 3.27
**MCV (fL)**	59.12	58.95	58.95	54.45^**ab**^	58.68
	± 2.34	± 1.08	± 1.76	± 2.68	± 2.03
**MCH (pg)**	19.9	18.45^**a**^	18.10^**a**^	17.33^**a**^	18.27^**a**^
	± 0.63	± 1.04	± 0.21	± 0.38	± 0.32
**MCHC (g/dL)**	34.46	33.74	30.71^**ab**^	30.25^**ab**^	32.38^**a**^
	± 0.56	± 0.50	± 0.24	± 0.42	± 1.58
**WBCs (10**^**3**^**/µL)**	13.24	9.81^**a**^	9.74^**a**^	9.06^**a**^	10.18^**a**^
	± 1.15	± 0.30	± 0.53	± 0.77	± 1.11
**PLts (10**^**3**^**/µL)**	644	698.3	766.1^**a**^	773.8^**a**^	736.2
	± 45.70	± 88.94	± 75.58	± 36.44	± 40.71

Data are presented as the mean ± SD. *CN*, control tap water; 10%, 50%, and 100%.EF, concentrations of MRI effluents; 100%.EF + AG, 100% effluents treated with Ca-alginate

a: Significant change in comparing different groups with the CN group (*p* < 0.05)

b: Significant change in comparing EF groups with 100%.EF + AG group (*p* < 0.05)

It should be mentioned that the obtained results showed nonsignificant difference between the treated EF group (100%.EF + AG) and the control one in most investigated parameters, except GST activity, GH levels, MCH, MCHC, and WBCs count.

#### Histological studies

##### The liver sections

**Fig. 5:**
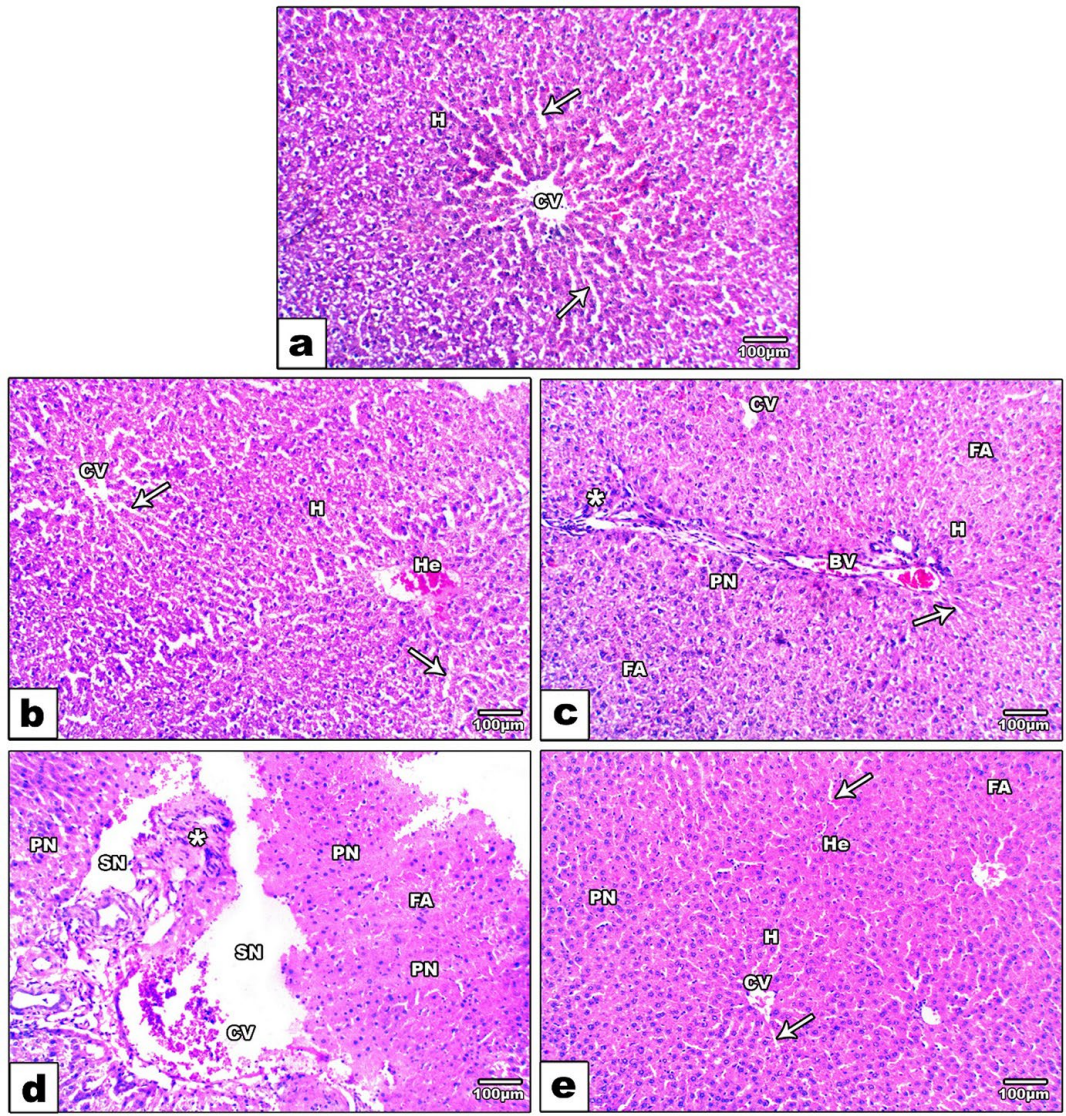
Photomicrographs of histological sections of the liver stained by H&E showing a control group (**a**) with normal central vein (CV), hepatocytes (H) and blood sinusoids (arrow). 10%.EF group (**b**) showing a relatively normal central vein (CV), hepatocytes (H) with distinct hepatic strands, blood sinusoids (arrow), and little hemorrhage (He). 50%EF group (**c**) showed foamy areas (FA) with elongated blood vessels (BV), cellular infiltration (star), pyknotic nuclei (PN), indistinct central veins (CV) and blood sinusoids (arrow). 100%. EF group (**d**) showing damaged central veins (CV), foaming area (FA) without distinct blood sinusoids, cellular infiltration (star), severe necrosis (SN), and pyknotic nuclei (PN). 100%. EF + AG group (**e**) showing improvement in liver architecture represented in appearance of central veins (CV), hepatocytes (H), with relatively clear blood sinusoids (arrow), little hemorrhage (He), and pyknotic nuclei (PN)

Histopathological investigations in the livers of different groups showed normal liver architecture with distinct hepatocytes consisting of hepatic stands and blood sinusoids surrounding intact central veins (Fig. 5a). The rat group received 10%EF showed little alterations represented in some hemorrhages, and slightly indistinct hepatocytes, blood sinusoids, and central veins in some areas (Fig. 5b). However, the liver sections of rats that received 50%EF appeared in foamy areas with elongated blood vessels, cellular infiltration, and disappearance of most hepatic strands and blood sinusoids (Fig. 5c). Moreover, consumption of 100%EF resulted in severe damage to the liver architecture represented in damaged central veins, and highly degenerated hepatocytes appeared as foamy areas with severe necrosis, cellular infiltration, and pyknotic nuclei (Fig. 5d). On the other hand, the group treated with Ca-alginate (100%Ef + AG) showed an obvious improvement in liver structure, where hepatocytes started to appear with central vein, hepatic strands, and blood sinusoids, decreased necrotic and foamy areas, little hemorrhage, and pyknotic nuclei (Fig. 5e).

##### The thyroid gland sections

**Fig. 6:**
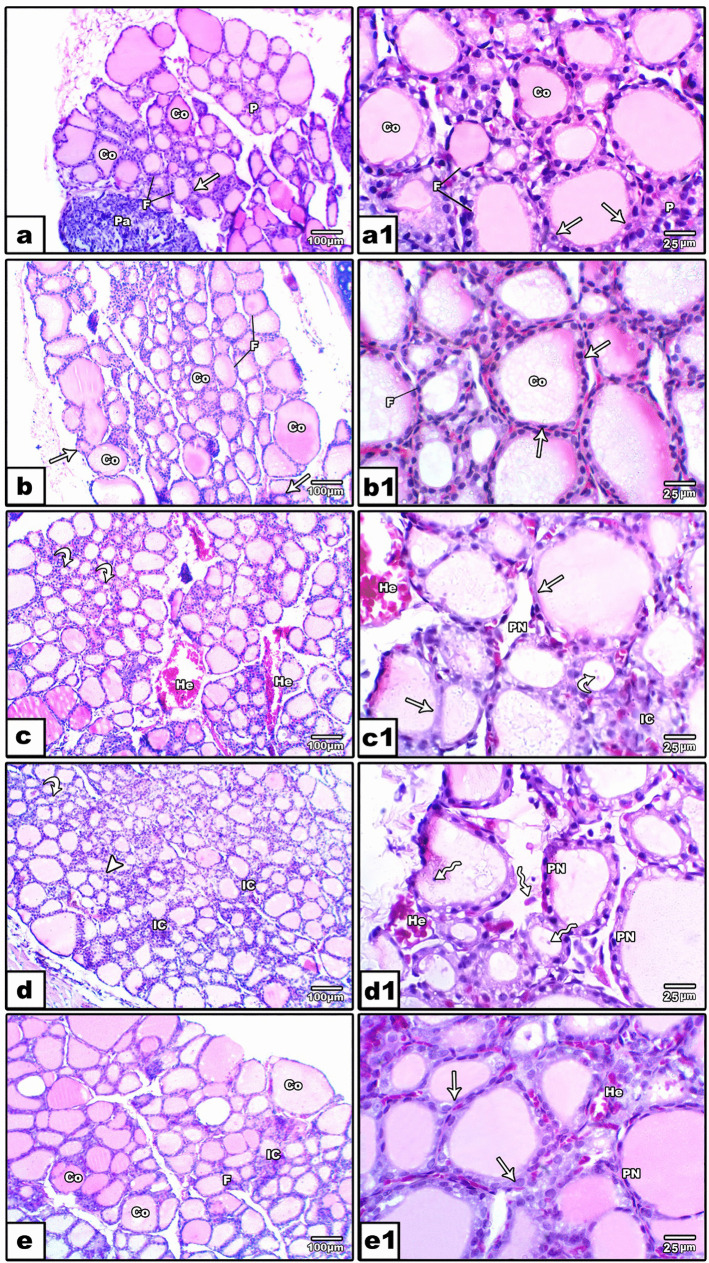
Photomicrographs of histological sections of the thyroid gland stained with H&E. Control group (**a**, **a**1) showing follicles (F), parafollicular cells (P), follicular epithelium lined with squamous to columnar cells (arrow), central lumen filled with colloid (CO), and a part of parathyroid gland (Pa) appeared. In the 10%EF group (**b**, **b**1) showing small retention of lobular architecture, follicular cells (F) filled with colloid (CO), and follicular epithelial cells containing flattened nuclei (arrow). The 50%EF group (**c**, **c**1) showed the formation of microfollicles (curved arrow), pale colloid (CO), hemorrhage (He), flatness of follicular epithelium (arrow), and the appearance of pyknotic nuclei (PN). In the 100%EF group (**d**, **d**1) numerous microfollicles (curved arrow), absence of colloid (CO) in most follicles, hyperplastic epithelium of follicular cells (arrowhead), inflammatory cells (IC), presence of migrated and isolated follicular cells (zigzag arrow), hemorrhage (He), and pyknotic nucleus (PN) were observed. For 100%EF + AG group (**e**, **e**1) follicular cells of various sizes (F) with lumen filled mostly with colloids (CO), hemorrhage (He), and inflammatory cells (IC) were observed and the nuclei of the follicular epithelium appeared rounded (arrow) with some pyknotic cells (PN)

The normal structure of the thyroid gland was represented in the control group, where parenchymal cells appeared in two types: follicular and parafollicular cells. Follicular cells (thyroid follicles) of various sizes lining a central colloid-filled lumen-stained pink (Fig. 6a) were observed. The follicular cell line ranged from squamous to low columnar epithelium with ovoid to round shaped nuclei. In addition, parafollicular cells were located on the periphery of the follicles and were not distributed in the interfollicular connective tissue (Fig. 6a1). Moreover, the 10%Ef group showed some retention in the lobular architecture of the thyroid gland but maintained its normal structure with a central filled lumen with colloid (Fig. 6b). However, the follicular epithelium showed some flatness in addition to the appearance of atrophied follicles (Fig. 6b1). Histological investigation of the thyroid gland in the 50%Ef group revealed multifocal migration of follicular epithelium cells forming several microfollicles in addition to hemorrhage between follicles (Fig. 6c). Additionally, the appearance of some follicular cells with pyknotic nuclei and the other showed large nuclei with a fine chromatin pattern (Fig. 6c1). Furthermore, the exposure to 100%.Ef group showed more thyroid disorders represented by the formation of more microfollicles with reduced lumen and colloid. The appearance of hyperplastic follicular cells with large nuclei and inflammatory cells was in between (Fig. 6d). Some follicles were also shown with projected papilloma in the lumen, and the latter was commonly paler than the control group (Figs. 6d1). On the other hand, treatment of 100% effluent with Ca-alginate (100%EF + AG) group showed an obvious improvement in the thyroid gland structure. This was represented by variations of follicular size surrounding the lumen filled with notable amounts of colloids and decreased hyperplasia, inflammatory cells, and hemorrhage (Figs. 6e). High power magnification showed follicular epithelium with some pyknotic nuclei (Fig. 6e1).

## Discussion

### Ecological studies

The direct discharge of untreated industrial effluents into drains, canals, or rivers worsens the problem of water pollution. The suitability of water used from drains, canals for irrigation purposes, and watering livestock was taken into consideration in this study. Estimation of certain environmental factors, such as pH, EC, TDS, and anionic and cationic components of water, plays an important role in water evaluation (Abugu et al., 2021; Islam et al., 2020; Sehlaoui et al., 2020).

The proficiency of water to pass out an electric current is known as electrical conductivity (EC) (WHO, 2008). The EC value is usually related to TDS or to the water mineral content (Reddy, 2013). The present data showed EC and TDS values in untreated MRI effluents within FAO irrigation limits, but both exceeded the WHO drinking water limits. Kaur et al. (2021) agreed with the present results, but attributed the elevated EC values to the increasing load of pollution either from agricultural runoff, sewage discharges, or industrial discharges, as seen in the current study. However, Safari et al. (2021) attributed higher levels of TDS to toxic solid materials that may be included in industrial effluents. Kaur et al. (2021) related the increase in water TDS to different types of minerals and organic substances. The present study also exhibited concentrations of anions and cations in the order of Cl^−^ > Na^+^  > SO_4_^−−^ > HCO_3_^−^ > Ca^++^  > Mg^++^  > K^+^, where levels of anions (Cl^−^ and SO_4_^− −^) and cations (Na^+^ and K^+^) components in MRI effluent samples were higher than the WHO and FAO limits for drinking and irrigation water. Tariq et al. (2020) reflected this to the discharge of high salt effluent concentrations. However, Safari et al. (2021) mentioned that atmospheric deposition, fertilizers, and oxidation of sulfur compounds by bacteria are the main sources of sulfate in water.

Moreover, the evaluation of water quality for irrigation purposes depends on its ionic composition (Sehlaoui et al., 2020). The concentrations of Na^+^, Ca^++^, Mg^++^, and HCO_3_^−^ in MRI effluents in this study were used to determine the quality of effluents for irrigation through detecting SAR, KR, Na%, PI, and MAR. Obtained results showed unsuitability of MRI effluents for irrigation purposes due to high values of SAR, KR, Na%, and PI. These results agreed with Veena and Satheeshkuamar (2018) who indicated that SAR studies the ion exchange reaction in the soil, where high SAR values represent serious physical problems lead to difficult absorption of water by plants. Reddy et al. (2013) illustrated that high SAR value gives the soil compactness and impervious features. Furthermore, KR values were recorded above 1, suggesting unsuitability for irrigation due to alkali hazards (Singh et al., 2020). On the other hand, Obasi et al. (2021) attributed the increase in Na% to the non-occurrence and subsequent nondissolution of sodic minerals. However, Veena and Satheeshkuamar (2018) related the high Na% in water to chemical fertilizers that are not applied here at the sampling area of discharge point of MRI effluents. The increase in Na% in irrigation water was reported to reduce soil permeability (Obasi et al., 2021). Besides, PI is another factor that affect soil permeability and regulate the water movement and nutrients uptake by plants (Abugu et al., 2021). The obtained results showed high PI values in MRI effluents that lies in the unsatisfied category. This was in agreement with Obasi et al. (2021) who attributed this to mining distrct.

On the other hand, Islam et al. (2020) considered elevated Cl^−^ concentrations as an indicator of high organic wastes of industrial origin. This may explain the high COD values obtained in the present study, suggesting that MRI effluents were polluted with a significant amount of organic matter discharged from the MRI company, which may be related to the manufacture of organic compounds such as formalin and urea. In agreement with these foundations, Traiq et al. (2020) attributed elevated COD levels to industrial sources. However, Kaur et al. (2021) correlated elevated COD values to the presence of both oxidizable organic and inorganic pollutants in industrial effluent indicating that a high COD value is indicative of water quality deterioration. Actually, this was achieved in the present study where data showed an increase in heavy metals (Cd, Co, Fe, Mn, Ni, Pb, and Zn) in MRI effluent samples above WHO and/or FAO limits. This may be because the MRI company is famous for producing foundry resins. The process of manufacturing these resins passes through different steps and reactions in which salts of divalent ions of Cd, Co, Fe, Mn, Ni, Pb, and Zn are used as catalysts (Mhamane et al., 2018). In this process, acidification through the addition of acids such as mineral acids is included (Pelit et al., 2019). This may explain the decreased pH value of effluent samples which was lower than the WHO and FAO limits for drinking and irrigation purposes, respectively. In addition, Kalsom et al. (2020) revealed that the acidic constituents of wastewater can be transformed into acidic compounds leading to a lower pH-value. This can be achieved in this study, as the MRI company is famous to produce chemicals such as formalin and urea that give effluents acidity.

Bioremediation of MRI effluent samples using Ca-alginate derived from brown algae was achieved in this study. The obtained data showed that the treatment of effluent samples for 5 h using Ca-alginate beads showed a significant decrease in heavy metal concentrations compared to untreated effluents. Zabochnicka-Swiatek and Krzywonos (2014) explained the mechanism of metal biosorption by microorganisms as the macromolecules of the cell wall structure, such as polysaccharides and proteins, have a negatively charged functional groups such as carboxyl, sulfhydryl, carbonyl, and hydroxy groups. These functional groups could gravitate positively charged metals found in a solution through the adsorption process (Chen et al., 2021). Moreover, Refaay et al. (2022) attributed the adsorption capacity of alginate to the richness with carboxyl groups, where calcium ions could be replaced by hydrogen ions on the carboxylic acid groups of the adjacent chains, forming a polymetric matrix of Ca-alginate distinguished by excellent pollutant capability through passive adsorption between metal ions and binding sites on the molecular structure (Tiwari & Kathane, 2013; Bilal & Iqbal, 2019; Aslam et al., 2021).

Moreover, the current data showed significantly positively correlated elemental pairs of effluent samples. This agreed with Tariq et al. (2020), suggesting that metals in industrial wastewater may have the same source or chemical phenomenon.

### Animal studies

In a process mimicking young livestock exposed to different concentrations of MRI effluents at different sites, the present study was concerned with possible hazards in young rats that received different effluent concentrations daily for 11 weeks. Metal bioaccumulation in the rat liver is of great concern due to its high sensitivity in the mammalian system to chemical toxicity. The obtained results showed that Fe, Mn, Zn, Pb, and Cd were the most accumulated metals in the liver depending on the effluent concentration (10%, 50%, and 100%) compared to the control group. This may be due to the absorbance of metals by intestinal cells that pass through the blood to the liver for the detoxification process by metallothionein protein synthesized in the liver (Adoeoye et al., 2015). Bioaccumulation of these heavy metals and organic compounds (not analyzed) may act in synergy and/or antagonism to induce several health alternations in a variety of body tissues and organs. The present results showed a significant increase in serum ALT and AST activities and TB concentration in the effluent groups compared to the control. Kanwar and Kowdly (2014) attributed this to the presence of Fe, although it is an essential element in the body, in excess, it could break the liver barrier, leading to discharge of cell content to the bloodstream. Osuala et al. (2014) added that Zn can also cause irritation in liver cells and decrease intracellular liver enzymes. Moreover, rough measures of protein status reflect major functional changes in the liver. The obtained data showed significant decrease in serum total protein and albumin contents depending on effluents concentrations compared to control group. Talkhan et al. (2016) agreed with these results and related exposure to heavy metals to reduced protein content by impairing protein metabolism. Therefore, the increased heavy metals and possibly unanalyzed organic constituents of MRI effluents could be a source of reactive oxygen species (ROS) that might disturb the oxidant/antioxidant defense system in the body. This was clearly observed in this study through the significantly elevated levels of liver MDA and PC in all effluent groups referring to lipid peroxidation and protein oxidation in liver cells. Hamdy et al. (2018) agreed with this result, where extreme generation of free radicals after exposure to Cd was the main cause of MDA and PC increases. In parallel, the obtained data showed a significant decrease in the liver SOD, CAT, and GST activities and GSH content in the effluent groups compared to the control group. The antioxidants SOD and CAT are the first line for body defense against toxicity. These enzymes were reported to be decreased due to excessive production of ROS induced by metal exposure (Hamdy et al., 2018). Shen et al. (2020) explained this through an in vivo study, where Pb and Cd can be combined with reductive sulfhydryl groups to antagonize the reducing effect of SOD and GSH and weaken the ability of organisms to oxidize and metabolize lipid products, leading to enhanced lipid peroxidation. Moreover, Balali-Mood et al. (2021) revealed that exposure to Cd could knock out the metallothionein gene which resulted in decreased CAT activity. Furthermore, in the literature, there are many conflicting reports on the effect of metals on the activity of GST. However, Kar et al. (2015) attributed the decrease in GST to exposure to heavy metals such as Cd which leads to the formation of transition complexes with protein enzymes and consequently inhibits the activity of antioxidant enzymes.

In support of this hypothesis, histological investigations in the livers of the investigated groups showed severe damage in the effluent groups depending on the concentration compared to the control group. Ebokaiwe et al. (2018) attributed this to the direct and/or indirect effect of elevated ROS, where the resulting lipid peroxidation can disrupt the structure of the liver. Kim et al. (2021) revealed that exposure to Pb could inhibit antioxidant enzymes, altering mitochondria and facilitating the release of O_2_^−^. The latter can attack liver tissue and distort its structural integrity (Ebokaiwe et al., 2018).

On the other hand, exposure to chemical pollutants including heavy metals may be related to alterations in the hypothalamic-pituitary-thyroid axis, which affects the body’s growth, development, and energy metabolism (Lauretta et al., 2019). Indeed, the present study focused on animals of young age to follow the possible growth changes. The abovementioned effects of metals on the oxidative breakdown of energy sources, including protein oxidation and reduction in total protein content, may provide an indication of the effect of metals on the hypothalamus-pituitary-GH axis. In this regard, the present study also showed significantly decreased levels of GH dependent on metal concentrations. Takahashi (2017) and Pan et al. (2021) reported that GH can stimulate the liver to produce insulin-like growth factor 1 (IGF-1), an important hormone in childhood growth. Thus, IGF-1 levels could be decreased as a result of GH inhibition and liver damage. Consequently, this may explain the significant decrease in body weight and body weight gain especially in the 100%EF group, compared to the control. This decrease was significantly detected after six weeks of exposure to 100% MRI effluents. This agreed with Kenston et al. (2018), where no significant difference in body weight was recorded after four weeks of exposure to a heavy metal mixture. Zhu et al. (2014) attributed the decrease in body weight to progressively severe systematic toxemia due to drinking effluents.

In support of this foundation, the thyroid gland also plays a crucial role in body growth control (Yazbeck, 2012). This can be achieved by regulating the metabolism of substances such as proteins, carbohydrates, and fats, where thyroid hormones mainly promote oxidative breakdown for energy supply (Sinha et al., 2018). The present study showed a significant decrease in serum T3 and T4 thyroid hormones in effluent groups with elevated metal concentrations compared to the control group. It was reported that most of the circulating thyroid hormone T3 is created through extrathyroidal deiodination of T4 which mainly occurs in the liver depending on 5^/^-monodeiodinase (5^/^-D) (Germain et al., 2009). Matovic et al. (2015) illustrated that Cd and Pb can inhibit 5^/^-D activity by binding to the sulphydryl groups of this enzyme. Balali-Mood et al. (2021) related the decrease in T4 levels to metal exposure, such as Cd and Pb, which influence the production and/or secretion of T4 by follicular cells. This probability was supported in this study by histological examination of the thyroid gland, where follicular cells collapsed and disintegrated with the lack of colloids, hyperplasia, damaged and/or reduced number of cell organelles, especially in the 50% and 100% Ef groups. This was incompatible with Elroghy and Yassien (2020), who explained that Cd could accumulate in the mitochondria of thyroid follicular epithelial cells and inhibit the synthesis and release of thyroid hormones, causing severe deterioration in both the structure and function of the thyroid gland. In parallel, the obtained data showed significantly increased levels of serum TSH in the effluent groups compared to the control. Yang and Ma (2021) explained the increase in TSH to stimulate thyroid cells to secrete T3 and T4 hormones through a negative feedback mechanism.

For complete assessment of health status in investigated rat groups, examination of blood parameters was achieved to illustrate its nutritional and pathological role. The results showed a significant decrease in most hematological indices (RBCs count, Hb content, Hct%, MCV, MCH, MCHC, and WBCs count), especially at 100%.EF group compared to control. David et al. (2020) attributed this decrease to the generated ROS from metals that disrupt hematopoiesis in the bone marrow.

Bioremediation using algae has been successfully used as a biosorbent for heavy metal detoxification (Bilal et al., 2018; Hamdy et al., 2018). The treatment of 100%EF with Ca-alginate (100%EF + AG) group showed significantly decreased accumulation of the studied heavy metals in the rat liver compared to different effluent groups. This result exactly referred to the successful role of Ca-alginate as a biosorbent for heavy metal detoxification. This was significantly clear with decreased oxidative stress parameters (MDA and PC) and increased antioxidants (SOD, CAT, GSH, GST) along with an amelioration in liver function represented by decreased ALT, AST,TB, increased TP and Alb, analyzed hormones (increased GH, T3, T4 and decreased TSH), and increased hematological indices (RBCs count, Hb content, Hct%, MCV, MCH, MCHC, and WBCs count). Moreover, histological alterations induced by drinking treated effluents (100%EF + AG) group showed clear improvement in both the structure of the liver and thyroid gland compared with the effluent groups. Hamdy et al. (2018) attributed this improvement to the marked reduction in metallothionein levels after treatment of heavy metal polluted water with algae.

## Conclusion

This study illustrated high effluents concentration of most physicochemical parameters and heavy metals (Cd, Co, Fe, Mn, Ni, Pb, and Zn) above the WHO and FAO limits, indicating the unsuitability of MRI untreated effluents for drinking or irrigation purposes. Treatment of effluents by using Ca-alginate beads for 5 h represented an effective and safe biosorbent for heavy metal decontamination and purification of effluents. However, non-significant changes in the physicochemical characteristics of effluents were observed with treatment. Moreover, in a process mimicking livestock exposure to polluted effluents at different sites, young rats exposed to different effluent concentrations showed significant increase in heavy metals concentration accumulated in liver of investigated groups. This was accompanied by significant disturbance in liver function and oxidative system along with disruptors in growth and thyroid hormones leading to growth retardation and body weight loss. All these foundations were supported by histopathological investigations in the liver and thyroid gland. This may indicate that closer villages to the MRI company, greater risks will be found, whether in terms of usage in irrigation or for watering livestock. However, rats received treated effluents with Ca-alginate beads showed significant amelioration in different investigated parameters compared to effluent groups. Nevertheless, Ca-alginate beads should be applied to MRI effluents for heavy metals biosorption, while further studies are needed to improve the physicochemical characteristics of effluents.

## Data Availability

All data used during the study appear in the submitted article and are available.
